# A statewide baseline inventory of evidence-based fall prevention programs for older adults

**DOI:** 10.1186/s40621-015-0046-3

**Published:** 2015-06-11

**Authors:** Jonathan Howland, Nicole J Treadway, Alyssa A Taylor, Elizabeth W Peterson

**Affiliations:** 1 Department of Emergency Medicine, Injury Prevention Center at Boston Medical Center, One Boston Medical Center Place, Boston Medical Center, Dowling 1 South, Boston, MA 02118 USA; 2 Department of Occupational Therapy, University of Illinois at Chicago, 919 West Taylor Street, Chicago, IL 60612 USA

**Keywords:** Falls, Injury prevention, Community-based, Intervention, Survey, Cross-sectional study

## Abstract

**Background:**

The Massachusetts (MA) Department of Public Health engaged the Injury Prevention Center at Boston Medical Center to develop a statewide baseline (2012) inventory of evidence-based (EB) community falls prevention programs for community-dwelling older adults.

**Methods:**

A web-based survey of organizations (*n* = 825) serving older adults was deployed in two parts. The Directors’ survey determined if a falls prevention program had been offered in 2012, the salience (rating of importance) of falls prevention for the organization, and intention to offer future falls prevention programming. A falls prevention program offered in 2012 triggered a second survey of Director-designated Coordinators to obtain information on programs’ dates and locations. For the last program offered, data were collected on the number of participants, the training and occupations of program facilitators, and program funding. The last programs served as a cross-sectional sample of all programs offered during 2012.

**Results:**

Response rates were 55 % (*N* = 457) and 86 % (*N* = 112) for the Directors’ and Coordinators’ surveys, respectively. The mean salience score for falls prevention was 3.68, on a 1–5 (most salient) scale; 12 % of respondents indicated offering ≥1 evidence-based program during 2012. We documented 107 EB programs, the majority of which (83 %) were offered by public agencies that serve older adults.

**Conclusions:**

Infrastructure for deployment of EB falls prevention programs is developing in MA, despite the absence of institutionalized funding, legislative mandates, widespread referrals from healthcare providers, or health insurance reimbursement.

## Background

Falls are significant and costly public health problems that affect millions of older adults nationwide. At least 30 % of those ages 65 and older experience at least one fall each year and half of these fall repeatedly (Stevens et al. 2006). In Massachusetts, as elsewhere, falls are the leading cause of injury-related deaths and non-fatal injuries among older adults (Massachusetts Department of Public Health 2014). Although fall-related death rates are lower in Massachusetts than in the USA as a whole, rates are increasing in both the state and the nation (Massachusetts Department of Public Health 2014). In 2010, fall-related injuries caused 434 deaths among Massachusetts older adults, 21,375 hospital stays, and 40,091 emergency department visits (Massachusetts Department of Public Health 2014).

The 2010 Massachusetts Behavioral Risk Factor Survey indicated that 35 % of older adults who experienced a fall in the prior 3 months sought medical attention and/or restricted activity for at least 1 day (Massachusetts Department of Public Health 2014). Of the Massachusetts older adults hospitalized for fall injuries in 2010, 20 % had traumatic brain injury and 10 % had hip or other femur fractures (Massachusetts Department of Public Health 2014). Non-fatal fall injuries are associated with decreased quality of life, lower functioning, and increased healthcare utilization (Sterling et al. 2001).

In 2010, in Massachusetts, costs attributable to falls were $512 million for inpatient care, $100 million for emergency room visits, and $19 million for observation hospital stays, a total of $631 million in direct medical care expenditures (Massachusetts Department of Public Health 2014).

Several decades of research on falls prevention have yielded relatively low cost, low-tech interventions that are evidence-based (EB) for falls prevention (Gillespie et al. 2012). These programs are increasingly deployed throughout Massachusetts and the nation. Falls prevention programs may eventually be integrated with the healthcare system as physicians become more engaged in falls risk assessment for their older patients, older adults become more aware that falls risk can be reduced, and when public and private healthcare insurers expand reimbursement for community-based falls prevention programming.

On behalf of the Massachusetts Commission on Falls Prevention (MCFP), the Massachusetts Department of Public Health (DPH) engaged the Injury Prevention Center (IPC) at Boston Medical Center to develop a statewide baseline inventory of EB community falls prevention programs for older adult Massachusetts residents living independently. A web-based survey was developed to count and characterize these programs during the index year 2012. The aim was to provide the MCFP, DPH, organizations that serve older adults, and other stakeholders with baseline data on statewide community-based falls prevention infrastructure. By identifying gaps in program availability by geography, facilitator training, and funding, the results could inform the development of strategies and resource allocation to enhance the state’s infrastructure for community-based falls prevention. To our knowledge, this is the first statewide inventory of EB falls prevention programs for community-dwelling older adults.

## Methods

### Operationalizing the variables

We could find no single list of EB falls prevention programs that served our purpose. Challenges to developing such a list included the fact that the lists of EB falls prevention programs published by the Centers for Disease Control and Prevention (CDC) (Stevens 2010) and the Administration on Aging (AOA; now the Administration on Community Living) (http//aoa.gov) differed, with some overlap. Agencies use different criteria to define “evidence-based.” Some exercise programs that do not necessarily target falls prevention have been found in clinical trials to reduce falls risk and therefore could be considered EB for falls prevention (Sherrington et al. 2011). Given these complexities, for the purposes of this project, we developed the following criteria for defining EB falls prevention programs for older adults:

1. Listed in the second edition of the CDC’s falls prevention program compendium
2. Listed as a third evidence tier falls prevention program by the AOA
3. Listed by the AOA as a first, second, or third evidence tier older adult exercise program that meets the criteria specified by the Sherrington et al. (2011) meta-analysis on exercise programs for falls prevention^7^. To satisfy the Sherrington criteria, the program must consist of at least 50 h of exercise, offer a balance component, and exclude a walking component (Sherrington et al. 2011)

We reviewed our approach with several experts based at public and private agencies that fund falls prevention programming and academic institutions. We acknowledge that as trials are conducted and published, the list of EB falls prevention programs will change with time, and that alternative methods might have yielded lists somewhat different from our own. Nonetheless, we believe that our method was appropriate for our purpose because it focused on programs endorsed by public funding agencies. The list of programs used for this study is available upon request from the corresponding author.

### Survey participants

We surveyed 825 organizations in 7 categories likely to have offered falls prevention programs to Massachusetts older adults during 2012. We surveyed a census of all of the organizations based upon the lists provided by interest groups and/or state registries. We did not sample because one aim of this project was to create an inventory of EB programs. Several types of organizations that might have also offered falls prevention programs were not surveyed, including hospitals, housing authorities, and municipal recreation departments. These groups were not surveyed because (1) falls prevention is secondary to their missions and (2) each group has a large number of members, which would have made it difficult to obtain reliable response rates.

Categories of organizations targeted by the survey were Area Agencies on Aging/Aging Services Access Points (AAA/ASAPs; *N* = 30), Councils on Aging (COAs; *N* = 347), YMCAs (*N* = 31, representing 82 branches), Community Health Centers (CHCs; *N* = 57), Assisting Living Residences (ALRs; *N* = 213), Home Health Agencies (HHAs; *N* = 123), and Community Action Agencies (CAAs; *N* = 24). For each category of organization, a tailored survey format was developed in collaboration with representatives of the organization (e.g., statewide associations). Tailoring included minor language changes in instructions, and for clinical organizations (e.g., Home Health Agencies), language that distinguish therapeutic services from community-based falls prevention programs. The data collected, however, were consistent across organization categories.

### Administration and content

A cover letter from the Massachusetts Secretary of Elder Affairs and the Commissioner of the Massachusetts Department of Public Health that explained the purpose of the survey was mailed to the directors/CEOs of targeted organizations.

The survey was administered on the web in two parts. The first part, the Directors’ survey, screened for organizations that conducted falls prevention programs in 2012. If a director indicated that his/her organization conducted or hosted a falls program (evidence or non-EB) in 2012, a second more detailed survey (Coordinators’ survey) was sent to a staff person (*N* = 148) designated by the director.

#### Directors’ survey

Directors were asked, “In 2012, did your organization provide or host a falls prevention program?” If directors answered “yes,” they were provided with a list consisting of EB programs and asked to check all that apply. If any falls prevention program was provided, directors were asked for the contact information of a designated coordinator who could provide program(s) details. Directors were asked, “Does your organization intend to directly provide or host a falls prevention program for your clients during 2012–2014?” Directors were also asked about the salience of falls prevention for their organization, using the following question: “Given the various services that comprise your organization’s mission, please indicate on a scale of 1 to 5 (1 = low priority and 5 = high priority) the priority of falls prevention programming for your clients.” The Directors’ survey took 5–10 min to complete.

#### Coordinators’ survey

This survey was sent to individuals designated by directors who indicated that at least one falls prevention program had been conducted or hosted by the organization in 2012. Designated coordinators were asked to confirm that a falls prevention program had been conducted or hosted in 2012, and, as in the Directors’ survey, they were asked to identify which programs were conducted using lists of EB programs and other related activities. For each EB program identified by coordinators, we collected the following information for each iteration thereof: (1) start date, (2) end date, (3) location, and (4) whether the program was offered directly or hosted by the organization. When programs began in 2011, but ended in 2012 or began in 2012 and ended in 2013, they were considered to have occurred in 2012 the index year.

In some cases, the director indicated that the organization had provided EB programs, but the coordinator indicated that this was not the case. When there was conflicting information regarding the offering of programs, we relied on the Coordinators’ data based on the assumption that coordinators were most apt to have accurate information. It is possible, however, that a few directors might have indicated that no falls prevention programs were offered by their organization during 2012, when there were in fact programs offered. In this case, the director would not designate an individual to complete the Coordinators’ survey, and if a program had been offered, it would not have been documented by this survey. Nonetheless, conflict in information provided by directors and coordinators was relatively rare, and thus, we assume that error in collected data was infrequent.

#### Cross-sectional sample

We asked a series of questions related specifically to the last EB program offered. This served as a cross-sectional sample of the characteristics of all documented EB programs, without having to collect data on all programs. Although the last programs might differ in some respects with the preceding programs, we believe that the characteristics described below generally represent the characteristics of the EB programs we inventoried.

For the last EB program delivered in 2012, the Coordinators’ survey asked about (1) the number of participants enrolled in the program, (2) the number of participants completing 80 % of the program, (3) whether the programs were led by lay people or human services professionals. (Lay individuals are those with no formal human services background, while professionals are those with human services credentials.), (4) whether facilitators had received training for the programs they led, (5) the professional background of the facilitators, (6) information about how the program was funded, and (7) whether fees were charged for program participation.

Qualtrics™ online survey software was used to develop and deliver both the Directors’ and Coordinators’ surveys.

### Data analysis

Our data are descriptive. Much of it is aggregated across organizational categories. When relevant, however, some data are presented by organizational categories. The number of older adults served was calculated by averaging the reported number of participants for the last programs and multiplying this by the overall number of EB programs reported by responding organizations.

### Feedback

At the conclusion of the survey for each category of the organization, we prepared a report for the DPH and MFPC and sent this organization specific report to the directors of all surveyed organizations in the category.

### Human subjects

The study was reviewed by the Institutional Review Board at Boston Medical Center.

## Results

### Response rates

**Fig. 1:**
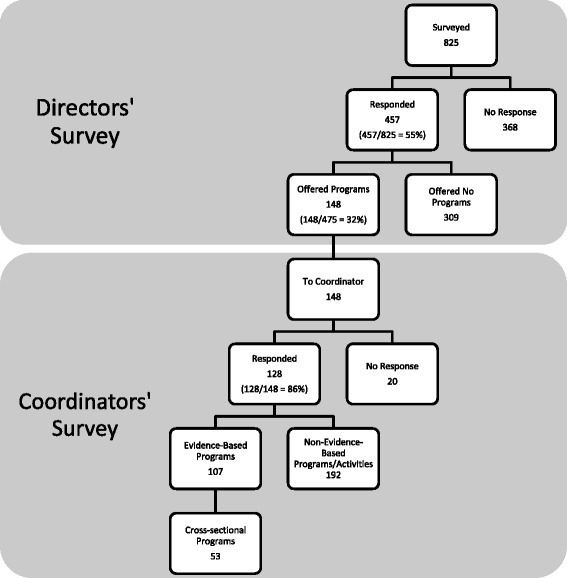
Responses to the Directors’ and Coordinators’ surveys

For each category of organization surveyed, there were two response rates: the rate for the Directors’ survey and the rate for the Coordinators’ survey. For the Directors’ survey, response rates varied across organization categories from 25 to 90 %; for the Coordinators’ survey, response rates varied from 67 to 100 %. The overall response rate was 55 % (457/825) for the Directors’ survey and 86 % (128/148) for the Coordinators’ survey. Figure 1 shows the responses to the Directors’ and Coordinators’ surveys. Table 1 presents response rates by type of organization.

**Table 1:** Response rates by organization

Organization type	Total number of organizations	Directors’ survey response	Directors reporting a falls prevention program	Coordinators’ survey response
		*n* (%)	*n* (%)	*n* (%)
AAA/ASAPs	30	27 (90 %)	14 (52 %)	14 (100 %)
Assisted Living Residence	213	91 (43 %)	39 (43 %)	26 (67 %)
Councils on Aging	347	229 (66 %)	93 (41 %)	67 (72 %)
Community Action Agencies	24	6 (25 %)	1 (17 %)	1 (100 %)
Community Health Centers	57	31 (54 %)	6 (19 %)	4 (67 %)
Home Health Agencies	125	51 (41 %)	7 (14 %)	6 (86 %)
YMCA	31 (representing 82 YMCA branches)	22 (71 %)	7 (32 %)	5 (71 %)
Totals	825	457 (55 %)	148 (32 %)	112 (76 %)

#### Directors’ survey

##### Falls prevention programming by organization type

AAA/ASAPs had the largest proportion (52 %) of organizations offering falls prevention programs in 2012, followed by COAs (43 %), ALRs (33 %), YMCAs (18 %), CAAs (17 %), and HHAs (12 %). None of the responding CHCs indicated that they had offered falls prevention programs. Overall, 32 % (148/457) of responding organizations reported offering falls prevention programming (EB or not) in 2012.

##### Intentions to offer falls prevention programming by organization type

ALRs had the largest proportion (79 %) indicating intentions to offer falls prevention programming during the years 2013–2014, followed by AAA/ASAPs (77 %), COAs (71 %), HHAs (61 %), YMCAs (45 %), CAAs (40 %), and CHCs (33 %). Overall, the number of organizations indicating intention to offer programs was double (310/148) the number that had actually offered programs during the 2012 baseline year.

##### Salience of falls prevention programming by organization type

Salience of falls prevention was highest among the directors of HHAs; HHA directors had a mean score of 4.46 on the salience scale, followed by directors of ALRs (4.26), COAs (3.82), AAA/ASAPs (3.55), YMCAs (3.44), CHCs (2.64), and CAAs (1.33). The weighted average of the salience rating for all responding directors was 3.68, on a scale of 1–5.

#### Coordinators’ survey

##### Evidence-based falls prevention programs offered in 2012

Overall, 12 % (53/457) of the organizations responding to our survey indicated that they had offered at least one EB falls prevention program during the index year 2012.

Of the 107 EB programs we documented, 37 % (40/107) were conducted by AAA/ASAPs, 46 % (50/107) by COAs, 11 % (12/107) by home health agencies, 5 % (5/107) by ALRs, and 1 % (1/107) by a YMCA. CHCs and Community Action Agencies provided no evidence-based programs.

##### Geographic distribution of evidence-based programs

Most of these programs were offered in the eastern portion of the state, with the greatest density in the Boston metro area.

#### Cross-sectional sample

##### Evidence-based falls prevention programming by organization type

Of the 53 last EB falls prevention programs offered by the organizations we surveyed, 90 % (47/53) were A Matter of Balance (Tennstedt et al. 1998), 9 % (5/53) were Tai Chi: Moving for Better Balance (Li et al. 2005), 1 % (1/53) was Simplified Tai Chi (Wolf et al. 1996), and 1 % (1/53) was Falls-HIT (Home Intervention Team) Program (Nikolaus and Bach 2000).

##### Estimated participation in evidence-based falls prevention programming

On average, 10.53 participants were served by each of the last programs conducted in 2012. Multiplying by 107, the total number of EB programs we documented yields a total of about 1127 participants.

##### Estimated completion rates for evidence-based falls prevention programs

Coordinators provided information on program completion rates for 79 % (42/53) of the EB programs offered. For the 2012 index year, Home Health Agencies and ALRs had the highest average completion rate (100 %), followed by COAs (88 %), YMCAs (85 %), and AAA/ASAPS (85 %).

##### Facilitator training for evidence-based falls prevention programs

Of the 53 last EB programs, information about facilitator training was provided for 81 % (43/53). Eighty-eight percent (41/43) of the falls prevention programs offered had at least one trained facilitator delivering the program.

##### Lay vs. professional facilitators

Of the 53 last EB programs, information about facilitator’s professional backgrounds was provided for 81 % (43/53). Forty-two percent (18/43) of the programs were facilitated by human services providers and 58 % (25/43) were facilitated by lay people.

##### Facilitators’ healthcare training

Of the 18 facilitators with backgrounds as human services providers, 39 % (7/18) were RNs. Physical Therapists and Social Workers made up 16 % (3/18) each. Case Managers, Education Specialists, Occupational Therapists, Personal Trainers, Health Promotions Managers, and Mental Health Counselors and other made up 6 % (1/18) each.

##### Funding for falls prevention programs

Of the 53 last EB programs, information on funding was provided for 81 % (43/53). Forty-four percent (19/43) of these programs were funded internally, 26 % (11/43) were funded externally, and 30 % (13/43) were funded by both internal and external funds.

##### Fees charged for falls prevention programs

Of the 53 last EB programs, information about fees charged to program participants was provided for 79 % (42/53). Eighty-one percent (34/42) indicated that they did not charge a fee for their falls prevention programs while 19 % (8/42) did.

## Discussion

Organizations established to serve older adults have taken the lead in providing EB community falls prevention programs. Specifically, AAAs, ASAPs, and COAs, all part of the service network funded by the Federal and State offices on aging, provided the majority of programs. Falls prevention programs are natural complements for elder services organizations, many of which already provide chronic disease self-care management programs, senior centers, transportation services, exercise programs, meals on wheels, and other support activities. Also, driving the dissemination of falls prevention programs is the fact that, increasingly, public agencies funding services for older adults are requiring that a proportion of these services be EB. Since several falls prevention programs are EB, conducting these programs helps local agencies meet funding criteria.

The most widely offered program by far was A Matter of Balance (MOB) (Tennstedt et al. 1998). Several factors might account for this. The program is well documented, and manuals and associated materials are available at relatively low cost. Though originally developed by researchers at Boston University to be led by healthcare professionals (Tennstedt et al. 1998), MaineHealth has developed a lay-led version of MOB and a train-the-trainer program that allows individuals who are not licensed healthcare providers to become master trainers who can in turn train program facilitators (coaches) (Healy et al. 2008). Consequently, a large pool of individuals (including retired older adults) is available as a source of volunteer program facilitators. This, combined with the proximity of MaineHealth (Portland, Maine) to Massachusetts, helps to provide the staffing required for program dissemination. In addition, the dissemination of MOB in Massachusetts has been supported by small grants from public and private organizations, most notably in Massachusetts, the Massachusetts Executive Office on Elder Affairs (Title III funds) and the Tufts Health Foundation.

Most of the EB programs we documented were led by facilitators who had been trained to lead the program. This likely reflects the fact that the majority of programs were MOB, training for which is both available and relatively inexpensive. As noted above, access to training accounts in part for the extensive deployment of MOB. This underscores the importance of accessible facilitator training for the development of falls prevention infrastructure.

Half of the EB programs conducted in the state during 2012 were internally funded. This likely reflects Title 3 funding from the Federal Administration of Aging, through the state Executive Office of Elder Affairs, to the AAAs, ASAPs, and COAs. Thus, many programs were offered with no fee. But, this finding also underscores the fact that falls prevention programs are inexpensive, relative to many healthcare interventions. Assuming that an organization has free access to space for conducting programs (e.g., senior centers, churches, schools), the per participant cost of MOB or Tai Chi could be relatively low. This has implications for the development of statewide falls prevention infrastructure, because it increases the likelihood that health insurers may eventually reimburse for these programs.

Also of note was our finding that completion rates for falls prevention programs were high, indicating that older adults value and/or enjoy participating in these programs, thus enhancing program effectiveness (as opposed to efficacy alone) and increasing demand for program deployment. However, thus far most participants have been self-selected. There is little or no data on uptake and completion rates among patients who are referred to falls prevention programs by their physicians.

It is likely that the number of community-based falls prevention programs will proliferate in Massachusetts and elsewhere. Our data suggest that for many organizations, the salience of falls prevention is high and the directors of many responding organizations indicated intentions to conduct future falls prevention programs. There is increasing awareness among healthcare providers and the public in general that many community-dwelling older adults can benefit from participation in falls prevention interventions. This trend should result in greater engagement of healthcare providers in falls prevention, which in turn will increase referrals and thus increase demand for community-based falls prevention programs. Healthcare provider awareness and engagement will be accelerated by the availability of instruments for assessing falls risk, such as the CDC’s STEADI toolkit (http://www.cdc.gov/STEADI) and reimbursement for falls risk assessment as part of the annual wellness visit reimbursed by Medicare under the Affordable Care Act. The evidence base for falls prevention strategies continues to grow as more trials are conducted, results published, and findings compiled in literature reviews and meta-analyses. These findings may eventually lead to increased reimbursement for EB community falls prevention programs by public and commercial health insurers.

It is likely that the results provide an incomplete inventory of Massachusetts EB falls prevention programs offered in 2012. The number of programs and the number of program participants are likely undercounted due to several factors:

- Not all organizations providing programs were surveyed (e.g., hospitals, housing authorities).
- Some surveyed organizations that provided programs may not have responded to the survey.
- The directors of some surveyed organizations that provided programs may not have been aware of, or may not have recalled, these programs, in which case a Coordinators’ survey would not have been sent.

Even if the undercount was on an order of 100 %, the number of older adults served would have been very small relative to the nearly one million Massachusetts residents 65 years or older.

Nonetheless, this study has several strengths. Our response rates, while modest for the Directors’ survey, were exceptional for the Coordinators’ survey. The study benefitted from the interest and cooperation of numerous public agencies and private associations representing the various categories of targeted organization. Our two-stage survey strategy allowed us to collect details about programs and their implementation, without compromising response rates.

## Conclusion

Our results indicate that infrastructure for the dissemination of evidence-based falls prevention programs, while limited, is developing in Massachusetts. Programs were offered throughout the state; the salience of falls prevention was high among directors and CEOs who responded to our survey; and most responding directors expressed intentions to offer future programs. Moreover, this dissemination occurred in the absence of institutionalized funding, organizational mandates, legislative policies, widespread referrals from healthcare providers, and health insurance reimbursement. In other words, for the most part, local organizations at the grass roots level have elected to offer fall prevention programs and market these directly to older adults.
